# Ovarian endometrioid carcinoma with a sex cord-like pattern: a morphological, immunohistochemical, and molecular analysis

**DOI:** 10.1007/s00428-024-03743-6

**Published:** 2024-02-29

**Authors:** Antonio Travaglino, Damiano Arciuolo, Angela Santoro, Caterina Fulgione, Alessia Piermattei, Manuela Martinelli, Maria Elisabetta Onori, Angelo Minucci, Antonio Raffone, Frediano Inzani, Gian Franco Zannoni

**Affiliations:** 1 Pathology Unit, Department of Woman and Child’s Health and Public Health Sciences, Fondazione Policlinico Universitario Agostino Gemelli IRCCS, Rome, Italy; 2 Pathology Unit, Department of Medicine and Technological Innovation, University of Insubria, Varese, Italy; 3 Pathology Institute, Catholic University of Sacred Heart, Rome, Italy; 4 Gynecology and Obstetrics Unit, Department of Neurociences, Reproductive Sciences and Dentistry, Federico II University of Naples, Naples, Italy; 5 Departmental Unit of Molecular and Genomic Diagnostics, Fondazione Policlinico Universitario A. Gemelli IRCCS, Rome, Italy; 6 Division of Gynecology and Human Reproduction Physiopathology, Department of Medical and Surgical Sciences (DIMEC), IRCCS Azienda Ospedaliero-Univeristaria Di Bologna, S. Orsola Hospital, University of Bologna, Bologna, Italy; 7 Anatomic Pathology Unit, Department of Molecular Medicine, University of Pavia and Fondazione IRCCS San Matteo Hospital, Pavia, Italy

**Keywords:** Endometrioid carcinoma, Sex cord, Sertoliform, TCGA, Immunohistochemistry, Genomic

## Abstract

Sex cord-like endometrioid carcinoma (SCLEC) is an uncommon entity which may constitute a diagnostic challenge. This study aimed to perform a clinicopathological, immunohistochemical, and molecular reappraisal of ovarian SCLEC. Consecutive ovarian SCLECs cases from a single institution were reviewed during a 13-year period. Twenty-three immunohistochemical markers were tested; 10 genes were analyzed by next-generation sequencing. Nine cases of ovarian SCLEC were identified. Mean patient age was 65.7 years; three cases showed extraovarian extension. Architectural pattern included sertoliform (*n* = 2), granulosa-like (*n* = 2), and mixed granulosa-like/sertoliform (*n* = 5). Eosinophilic changes accompanied by increased nuclear atypia were observed in four tumors. Endometrioid features (glands, squamous/morular differentiation) were observed in six cases. Most tumors were positive for cytokeratin-7 (8/9), EMA (9/9), estrogen and progesterone receptor (9/9), CD10 (7/9, including a luminal pattern reminiscent of mesonephric neoplasms), nuclear β-catenin (8/9), and CDX2 (8/9). A minority of cases showed block-type p16 pattern (2/9), PAX8-positivity (3/9), and non-diffuse positivity for WT1 (1/9), inhibin (1/9), chromogranin (1/9), and synaptophysin (2/9). All cases were negative for GATA3, TTF1, calretinin, and SF1. Ki67 range was 15–90%. Six cases showed *CTNNB1* exon 3 mutation. Eight cases were of “no specific molecular profile” (NSMP) and one was p53-abnormal. In conclusion, SCLECs frequently exhibit a mixed sertoliform/granulosa-like architecture and express epithelial markers, hormone receptors, nuclear β-catenin, and CDX2, with luminal CD10 positivity and *CTNNB1* mutations. PAX8 expression is often lost, while other mesonephric, sex cord, and neuroendocrine markers are negative.

## Introduction

Endometrioid carcinoma (EC) displays morphological heterogeneity with a wide range of architectural patterns, potentially constituting a diagnostic pitfall [1]. A clear example is offered by EC mimicking sex cord tumors, which are more commonly encountered in the ovary than in the endometrium. Such group includes sertoliform EC, characterized by solid and hollow tubules mimicking Sertoli/Sertoli-Leydig cell tumor [2–4]. However, EC can also mimic other sex cord tumors, in particular, granulosa cell tumor [2, 5], so we prefer to use the definition “sex cord-like EC” (SCLEC). The distinction between SCLEC and true sex cord tumors is based on both morphological and immunohistochemical features. Indeed, SCLEC often shows typical EC features such as squamous, mucinous or ciliated differentiation, a conventional EC component, or adjacent endometriosis and/or a seromucinous tumor [2–7]. The unconventional architecture of SCLEC can also mimic neuroendocrine tumors, mesonephric-like carcinoma, female adnexal tumor of probable adnexal origin (FATWO), and the recently described *STK11* andexal tumor [7, 8]. Immunohistochemistry appears therefore crucial for a correct diagnosis.

Despite its unusual morphology, SCLEC typically behaves as a low-grade tumor and shows excellent prognosis when confined to the ovary [2, 3]. Given its rarity, no data are available about the molecular background of SCLEC. Recent evidence suggests that ovarian EC can be stratified into four molecular prognostic groups by using the TCGA classification of endometrial carcinoma [9].

This study aimed to provide a clinicopathological, immunohistochemical, and molecular reappraisal of SCLEC.

## Materials and methods

All ovarian SCLECs diagnosed between January, 2010, and December, 2022, were retrieved from the digital database of our institution. Cases with available material for histological review and immunohistochemical and molecular analyses were included. Histological diagnosis was performed based on previously reported morphological and immunohistochemical criteria [2–7]. Histological and immunohistochemical slides were reviewed by a panel of five gynecological pathologists (AT, DA, AS, FI, and GFZ) to confirm the diagnosis of SCLEC.

### Histological and immunohistochemical methods

Histological and immunohistochemical procedures were performed as previously described [10, 11]. Antibodies against CK7 (clone RN7; ready to use; Leica), EMA (clone E29; 1:500; Dako), inhibin (clone MRQ-63; ready to use; Roche), calretinin (clone Dak-calret 1; 1:59; Dako), SF1 (clone EPR19744; 1:50; Abcam), chromogranin (clone LK2H10; ready to use; Roche), synaptophysin (clone SP11; ready to use; Roche), CD10 (clone 56C6; ready to use; Leica), GATA3 (clone L50-823; ready to use; Roche), TTF1 (clone SPT-24; ready to use; Leica), WT1 (clone WT49; ready to use; Leica), estrogen receptor (ER) (clone SP1; ready to use; Roche), progesterone receptor (PR) (clone 1E2; ready to use; Roche), β-catenin (clone 14; ready to use; Roche), PAX8 (clone EP331; ready to use; Roche), CDX2 (clone EPR 2764Y; ready to use; Roche), p16 (clone 6H12; ready to use; Leica), p53 (clone Do-7; ready to use; Leica), MLH1 (clone ESO5; ready to use; Leica), MSH2 (clone 79H11; ready to use; Leica), MSH6 (clone EP49; ready to use; Leica), PMS2 (clone EPS1; ready to use; Leica), PTEN (clone 6H2.1; ready to use; Agilent Dako), and Ki67 (clone 30–9; ready to use; Roche) were used. Leica Bond III (Leica Byosystems, Wetzlar, Germany), Ventana Benchmark Ultra (Roche Diagnostics, Basel, Switzerland), and Dako Omnis (Agilent Dako, Santa Clara, CA, USA) were used as automatized platforms for immunohistochemistry.

### Molecular analysis

Molecular analyses were performed as previously described [12]. The MagCore Genomic DNATissue Kit by MagCore HF16 Plus (Diatech Lab Line, Jesi, Italy) was used to extract DNA from formalin-fixed, paraffin-embedded tissue. Histological sections with a minimum neoplastic cellularity of 30% were selected. A Qubit dsDNA HS assay (Thermo Fisher Scientific, Waltham, MA, USA) was used to determine DNA concentration and quality.

Next-generation sequencing was performed on the Illumina MiSeq platform according to manufacturer’s standard protocol (Illumina Inc., San Diego, CA). The LYNCH-FAP Devyser Kit (Devyser, Hägersten, Sweden) was used to screen tumor samples for somatic variants of *POLE*, *POLD1*, *MLH1*, *MSH2*, *MSH6*, *PMS2*, *EPCAM*, *APC*, *MUTYH*, and *CTNNB1.*

The Amplicon Suite software (SmartSeq s.r.l., Novara, Italy) was used to process next-generation sequencing data.

## Results

### General features

Twelve cases of ovarian SCLEC diagnosed at our institution between 2010 and 2022 were identified. Out of these ones, two had no available material for histological review and immunohistochemical/molecular analyses and one was diagnosed as *STK11* adnexal tumor on histological review (confirmed by molecular analysis). Therefore, nine cases were finally included in our study.

Mean patient age was 65.7 years (range 50–89). Mean tumor diameter was 12.9 cm (range 7–18.5 cm). In six cases, the tumor was limited to one ovary at diagnosis; one case showed lymph node metastasis, one case showed bilateral ovarian and peritoneal involvement, while the remaining case showed peritoneal involvement with no contralateral ovarian involvement.

### Histology

**Fig. 1:**
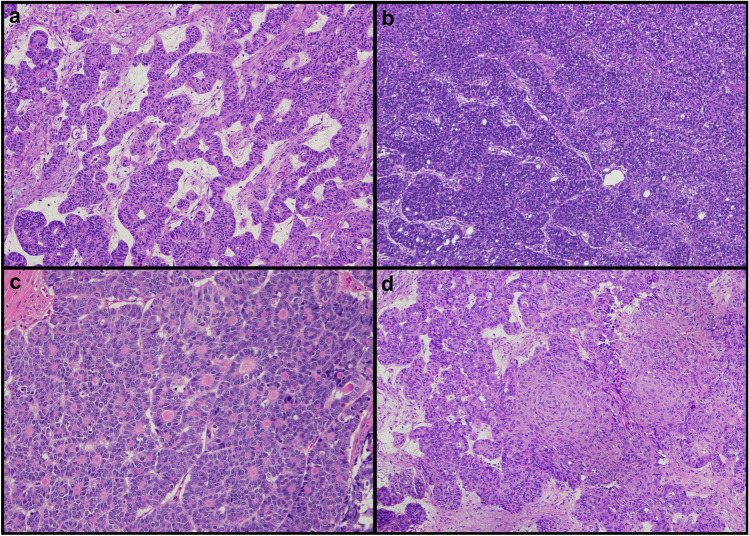
Morphological features of sex cord-like endometrioid carcinoma (SCLEC). **a** Sertoliform pattern consisting of anastomosing solid and hollow tubules. **b** Solid-trabecular architecture resembling granulosa cell tumor. **c** Microfollicular pattern. **d** Squamoid areas

**Fig. 2:**
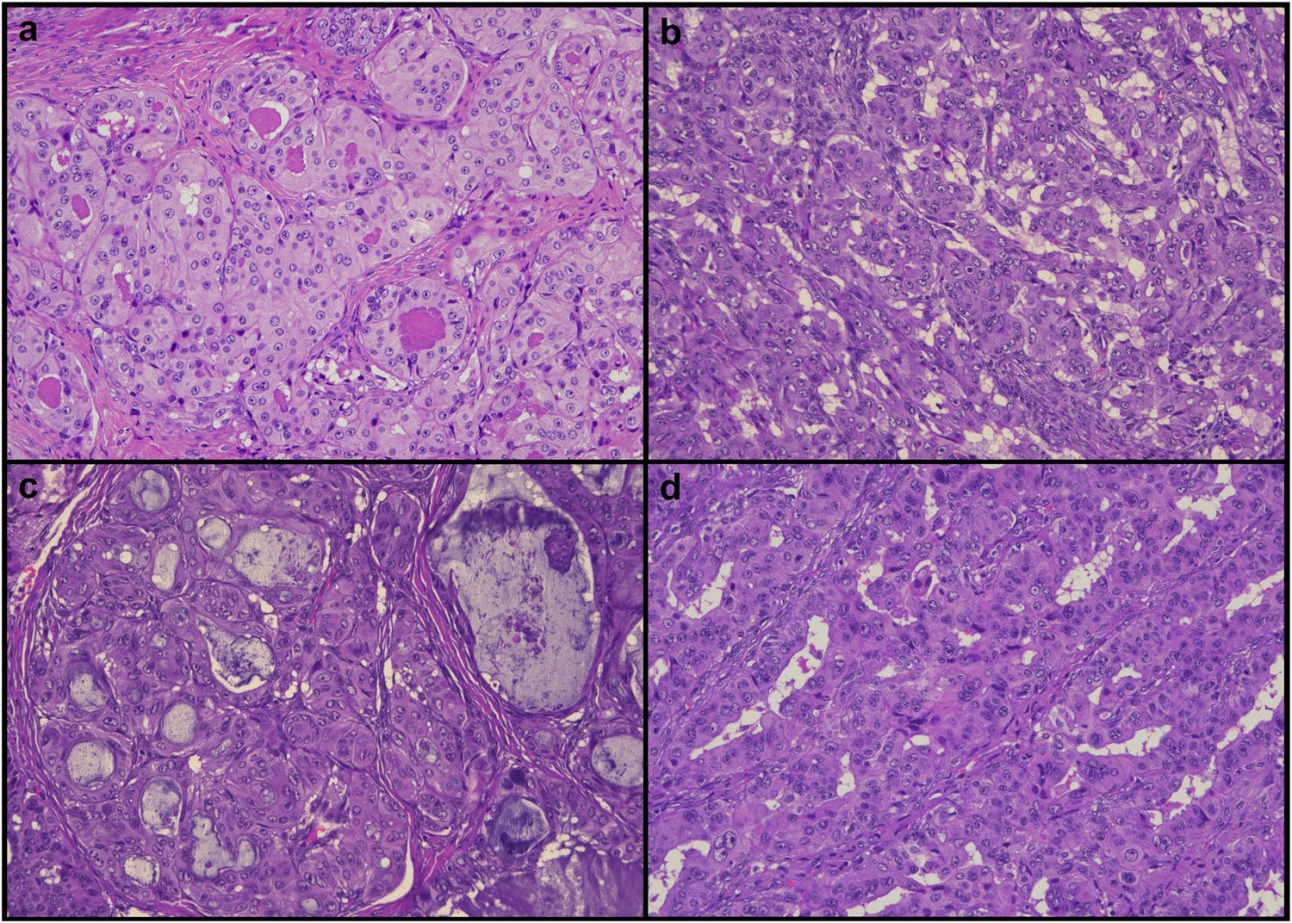
Cytoplasmic eosinophilia and nuclear pleomorphism in sex cord-like endometrioid carcinoma (SCLEC). **a** Tumor cells resembling Hürthle cells. **b** Trabecular pattern. **c** Mucinous features. **d** Serous-like atypia

Histologically, two tumors showed a sertoliform architecture (i.e., a combination of solid and hollow tubules), two cases showed a granulosa cell tumor-like architecture (i.e., solido-trabecular pattern with microfolliculi), and five cases showed a mixed sertoliform/granulosa-like features (Fig. 1a–c). An unequivocal EC glandular component was observed in three tumors. Squamous/morular differentiation was observed in four cases (Fig. 1d). All cases showed at least focally an eosinophilic luminal secretion. Five cases showed low-grade nuclei, with uniformly dark chromatin in two cases and variable chromatin (dark to dispersed) in three cases. One of these cases showed ovoidal-to-spindled nuclei. The remaining four cases showed areas with eosinophilic changes, enlarged nuclei, and evident nucleoli, which were associated with foci of squamoid and/or mucinous features; the eosinophilic changes focally showed nuclear pleomorphism potentially mimicking high-grade serous carcinoma (Fig. 2). No nuclear grooves were observed.

Clinicopathological features of the nine study cases are summarized in Table 1.

**Table 1:** Clinicopathological characteristics of the nine study cases

Case no	Age	Stage	Size	Appearance	Nuclear grade	Cytoplasm eosinophilia	Luminal secretion	Endometrioid features
1	50	Both ovaries Ovarian peritoneum	13 cm	Sertoliform	Low to moderate	No	Present (diffuse)	Morular metaplasia
2	64	Limited to the ovary	7 cm	Sertoliform and granulosa-like	Low	No	Present (focal)	None
3	69	Limited to the ovary	18.5 cm	Sertoliform and granulosa-like	Moderate	No	Present (diffuse)	None
4	89	Limited to the ovary	15 cm	Granulosa-like	Low	No	Present (zonal)	None
5	54	Limited to the ovary	6.5 cm	Sertoliform and granulosa-like	Moderate to high	Present (diffuse)	Present (diffuse)	Morular metaplasia
6	65	Limited to the ovary	13 cm	Sertoliform	Moderate to high	Present (zonal)	Present (focal)	Glandular component
7	52	Ovarian peritoneum	14 cm	Granulosa-like	Low	No	Present (zonal)	Squamous/morular metaplasia
8	74	LN metastasis	16 cm	Sertoliform and granulosa-like	Moderate to high	Present (diffuse)	Present (diffuse)	Glandular component; squamous/morular metaplasia
9	74	Limited to the ovary	17 cm	Sertoliform and granulosa-like	Moderate to high	Present (zonal)	Present (diffuse)	Glandular component

*ER*, estrogen receptor; *PR*, progesterone receptor; *MMR*, mismatch repair proteins; *foc*, focal; *zon*, zonal; *diff*, diffuse; *het*, heterogeneous; *wt*, wild type; *mut*, mutated

### Immunohistochemistry

**Fig. 3:**
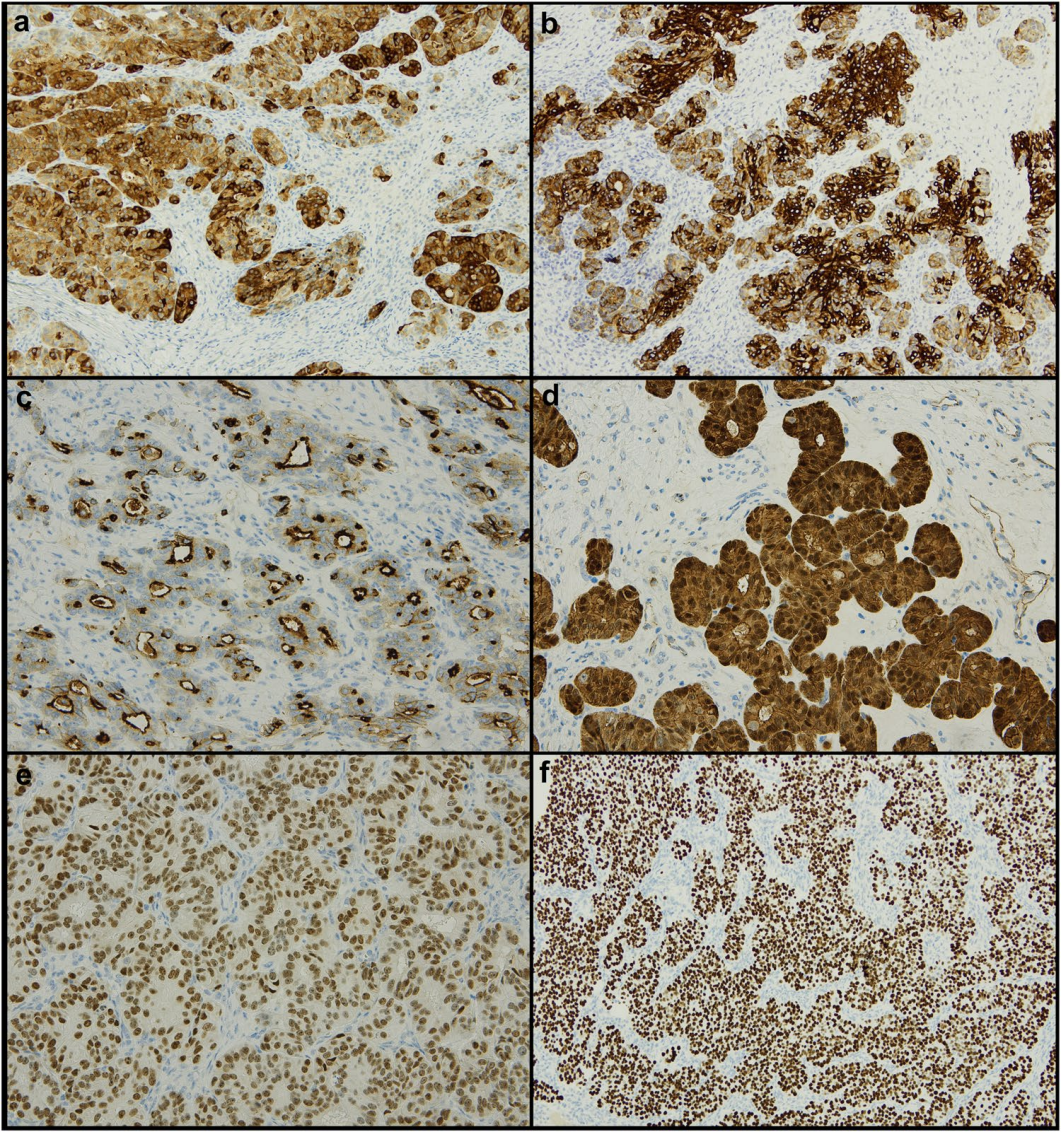
Immunohistochemical features of sex cord-like endometrioid carcinoma (SCLEC). **a** Diffuse positivity for EMA. **b** Diffuse positivity cytokeratin-7. **c** Luminal expression of CD10 resembling mesonephric neoplasms. **d** Diffuse nuclear accumulation of β-catenin. **e** Diffuse CDX2 expression. **f** One case was p53-abnormal

Immunohistochemically, all tumors were positive for EMA (Fig. 3a), with a strong and diffuse expression in five cases and zonal expression in four cases. Eight out of nine cases were positive for CK7 (Fig. 3b) (diffuse positivity in four cases and zonal positivity in four cases).

All tumors were negative for sex cord markers (inhibin, calretinin, SF1), except for one cases showing focal inhibin expression. Six out of nine tumors were completely negative for neuroendocrine markers; only one case was focally positive for chromogranin, while two cases showed focal and zonal positivity for synaptophysin.

CD10 was expressed in 7/9 tumors, with a diffuse positivity in six cases and a zonal positivity in one case; the expression pattern was heterogeneous but consistently included areas with luminal positivity reminiscent of mesonephric-like carcinoma (Fig. 3c). All tumors were negative for GATA3 and TTF1. PAX8 was diffusely expressed in only three cases, while it was negative in the remaining cases.

All cases showed moderate-to-strong positivity for ER and PR, with ER positivity ranging from 40 to 90% of tumor cells (mean 70%) and PR positivity ranging from 25 to 90% of tumor cells (mean 81.1%).

P16 expression was patchy in six cases, diffuse in two cases, and focal in one case. Ki67 labeling index ranged from 15 to 90%. Only one case showed WT1 expression (zonal).

Nuclear expression of β-catenin was found in seven cases (Fig. 3d), six of which with a diffuse pattern; the latter ones also showed CDX2 positivity (Fig. 3e).

Immunohistochemical findings are summarized in Table 2.

**Table 2:** Immunohistochemical and molecular characteristics of the nine study cases

Case no	CK7	EMA	PAX8	Inibin	Calretinin	SF1	ER	PR	Chromo	Synapto	GATA3	TTF1	WT1	CD10	β-cateinin (nuclear)	CDX2	Ki67	p16	p53	MMR	Mutations
1	-	+ (zon)	-	-	-	-	+ (90%)	+ (90%)	-	-	-	-	-	+ (diff)	+ (diff)	+ (diff)	18%	+ (zon)	wt	+	None
2	+ (diff)	+ (diff)	-	-	-	-	+ (40%)	+ (40%)	-	-	-	-	+ (zon)	+ (diff)	+ (diff)	+ (foc)	20%	+ (zon)	wt	+	CTNNB1: c.98C > T; p.Ser33Phe
3	+ (zon)	+ (zon)	-	-	-	-	+ (90%)	+ (90%)	-	+ (zon)	-	-	-	-	-	-	17%	+ (zon)	wt	+	None
4	+ (zon)	+ (zon)	-	-	-	-	+ (70%)	+ (90%)	-	-	-	-	-	-	+ (foc)	+ (zon)	30%	+ (zon)	wt	+	CTNNB1: c.134 > T; p.Ser45Phe
5	+ (diff)	+ (diff)	-	-	-	-	+ (75%)	+ (75%)	-	+ (foc)	-	-	-	+ (diff)	+ (diff)	+ (diff)	15%	+ (zon)	wt	+	CTNNB1: c.101G > T; p.Gly34Val
6	+ (diff)	+ (diff)	+	-	-	-	+ (50%)	+ (90%)	-	-	-	-	-	+ (diff)	+ (diff)	+ (zon)	15%	+ (foc)	wt	+	CTNNB1: c.101G > T; p.Gly34Val
7	+ (diff)	+ (diff)	-	+ (foc)	-	-	+ (60%)	+ (25%)	+ (foc)	-	-	-	-	+ (zon)	+ (diff)	+ (zon)	90%	+ (diff)	mut	+	None
8	+ (zon)	+ (diff)	+	-	-	-	+ (90%)	+ (70%)	-	-	-	-	-	+ (diff)	+ (diff)	+ (diff)	38%	+ (diff)	wt	+	CTNNB1: c.101G > T; p.Gly34Val
9	+ (zon)	+ (zon)	+	-	-	-	+ (65%)	+ (90%)	-	-	-	-	-	+ (diff)	+ (diff)	+ (diff)	Het (10 to 70%)	+ (zon)	wt	+	CTNNB1: c.101G > T; p.Gly34Val

### TCGA classification and molecular results

With regard to the TCGA classification, all tumors were MMR proficient and *POLE* wild type. One case showed aberrant overexpression of p53 and was therefore classified as p53-abnormal (Fig. 3f); the remaining cases were classified as NSMP. Five tumors showed mutation in the exon 3 of *CTNNB1*. No other pathogenic mutations were found.

TCGA classification and molecular findings are reported in Table 2.

## Discussion

Our study provided a clinicopathological, immunohistochemical, and molecular reappraisal of ovarian SCLEC. We found that most SCLECs show an admixture of sertoliform and granulosa-like architecture, highlighting the frequent nuclear β-catenin and CDX2 expression, luminal CD10 positivity, and *CTNNB1* mutations.

In agreement with the results of previous studies, patients with SCLEC in our series showed a highly variable age at diagnosis (range 50–89 years; mean 65.7). Such range overlaps with both conventional EC and adult granulosa cell tumor [13, 14], while it differs from Sertoli-Leydig cell tumor, which mostly occurs at a young age (mean age ~ 25 years) [15]; this may be helpful in differential diagnosis. Similar to conventional low-grade EC, most SCLEC cases show relatively favorable prognosis, with no extension beyond ovary [3]. However, a minority of cases may show advanced stage and/or aggressive behavior [16]. In our series, 3/8 cases showed extraovarian extension; one of these cases was p53-abnormal.

While previous studies on SCLEC mainly included sertoliform EC [2–4], in our series the granulosa cell tumor-like pattern and sertoliform pattern had similar frequencies. In 5/9 cases of our series and in most previously published cases, SCLEC showed low-grade features with no high nuclear pleomorphism [1, 2]; four cases showed eosinophilic changes with enlarged nuclei to a variable extent, with areas of serous-like pleomorphism. While striking pleomorphism might potentially represent a diagnostic pitfall, it was not diffusely present in any of the included cases. Interestingly, all and only the cases exhibiting cytoplasmic eosinophilia and enlarged nuclei harbored *CTNNB1* c.101G > T; p.Gly34Val. mutation. It is unclear whether this association is meaningful.

Given the wide range of adnexal tumors exhibiting a sex cord-like architecture [17], the presence of EC morphological features in SCLEC can be crucial for a correct diagnosis. While most previously published cases of SCLEC were accompanied by an overt EC glandular component [1, 2], the latter was present in only three cases of our series. Squamous/morular differentiation was observed in four cases. Remarkably, three cases lacked unequivocal EC features and were diagnosed by a combination of morphological, immunohistochemical, and molecular features; *STK11* alterations were also analyzed and excluded in these tumors. In ambiguous cases, immunohistochemistry may help identifying the case as a SCLEC by showing positivity for epithelial markers (CK7, EMA) and negativity for sex cord markers (inhibin, calretinin, SF1) [6–8]. In our series, all cases were positive for EMA and 8/9 for CK7, while only one case was focally positive for inhibin. An advanced age and the absence of hormonal manifestation would also favor SCLEC [2].

As previously described, the differential diagnosis of SCLEC is broad and includes not only sex cord tumors but also neuroendocrine, Wolffian/mesonephric-like neoplasms, and *STK11* adnexal tumors [7, 8]. Neuroendocrine tumors may be excluded based on negativity for neuroendocrine markers. In our series, only one case was focally positive for chromogranin and two were focally/zonally positive for synaptophysin (which is less specific) [18].

FATWO is a rare entity which may show overlapping morphology and immunophenotype with SCLEC. Unlike SCLEC, FATWO is mostly negative or focally positive for EMA and often shows a non-diffuse expression of sex cord markers; however, the immunophenotype of FATWO has been shown to be inconsistent. PAX8 is reportedly more common in SCLEC than in FATWO, but in our series only three SCLECs expressed PAX8. Moreover, upper mesonephric remnants (from which FATWO is thought to derive) are positive for PAX8 [8]. We have therefore some concerns about the accuracy of this marker in the differential diagnosis. Morphological features of FATWO that may be helpful in the differential diagnosis are para-tubal localization, multilobate architecture, expansile borders, bland nuclear features, and low mitotic index [8, 19].

Mesonephric-like carcinoma is an aggressive entity which exhibits a wide range of morphological patterns. The typical immunophenotype of mesonephric-like carcinoma includes CD10 luminal expression; PAX8, GATA3, and TTF1 nuclear positivity, negativity, or focal positivity for ER and PR; retained MMR expression; wild-type p53 pattern; and non-diffuse p16 expression [8, 17, 20]. In our series, most cases showed a luminal expression of CD10, which might raise the concern of a mesonephric-like carcinoma. However, all SCLECs in our series were negative for GATA3 and TTF1 and positive for ER and PR, allowing us to exclude mesonephric-like carcinoma. Moreover, 6/9 SCLECs were negative for PAX8.

*STK11* adnexal tumor is a recently described entity, previously included in the FATWO category. In most cases of our series, morphological and immunophenotypical features did not appear consistent with *STK11* adnexal tumors. In fact, the latter mostly show paratubal localization and a peculiar architecture characterized by interanastomosing cords and trabeculae immersed in a myxoid matrix, and basophilic rather than eosinophilic intraluminal secretion. Unlike our cases, most *STK11* adnexal tumors showed diffuse WT1 and calretinin expression and focal CK7 and CD10 positivity, while EMA is uncommonly expressed [8, 21]. In our series, we only tested *STK11* in two tumors which lacked obvious endometrioid features. This was useful to make a diagnosis of SCLEC by exclusion. We excluded another case of putative SCLEC from our study because it showed morphological and immunophenotypical features suggestive for *STK11* adnexal tumor on pathological review; molecular analysis confirmed the presence of *STK11* alteration in that case.

Another novelty of our study is the finding of nuclear β-catenin positivity in 8/9 SCLECs. The presence of nuclear β-catenin accumulation in SCLEC might suggest that it is involved in the development of sex cord-like features. In fact, ECs with nuclear β-catenin often show altered differentiation, such as morular metaplasia, “corded and hyalinized” pattern, and pilomatrix carcinoma-like morphology [22–25]. As observed in morular metaplasia, in our series nuclear β-catenin was accompanied by CDX2 positivity [26]. Since CDX2 is used as a marker of gastrointestinal differentiation [27–29], its expression may be a potential pitfall if the differential diagnosis includes a metastasis from an extraovarian site, especially if Müllerian marker PAX8 is negative. In these cases, the moderate-to-strong positivity for ER and PR, combined with the negativity for the breast epithelial marker GATA3, may suggest the gynecological origin of the neoplasm; however, a comprehensive immunohistochemical evaluation remains crucial for a correct diagnosis.

Other possible differential diagnoses may include malignant Brenner tumor (which, unlike SCLEC, is typically ER/PR-negative and GATA3-positive) and EC with a transitional-like pattern (another morphological variant of EC with seemingly no clinical significance, more common than SCLEC) [17, 30].

Our study also attempted to provide the first TCGA-based assessment of SCLEC. We found that 8/9 tumors were NSMP; such a finding could be expected, given the similarities between SCLEC and conventional low-grade EC. No case fell into the MMR-deficient or POLE-mutant group; in this regard, these molecular groups are less common in ovarian carcinoma than in endometrial carcinoma [9]. One case in our series was p53-abnormal; this case also showed diffuse p16 expression and a high proliferation index (Ki67 = 90%). A subsequent molecular analysis performed for treatment purposes (and not par of our study) showed BRCA mutation, leading us to consider a diagnosis of high-grade serous carcinoma; however, this was excluded based on the low-grade nuclear atypia, the presence of squamous and morular differentiation, and the endometrioid-type immunophenotype (negativity for WT1, loss of PTEN expression, nuclear β-catenin accumulation) [31]. This case was therefore considered a *bona fide* p53-abnormal SCLEC, highlighting the molecular heterogeneity of this entity. In our previous paper, we also described a case of a BRCA-mutant high-grade serous carcinoma with a sex cord-like pattern [32]; these cases suggest a possible association between BRCA alterations and a sex cord-like architecture in ovarian carcinomas.

Limitations of our study mainly include the low number of tested genes and the relatively small sample size, which preclude to draw conclusions about the molecular background and TCGA classification of SCLEC.

## Conclusion

SCLEC is an uncommon variant of EC which mimic sex cord tumors (mostly exhibiting mixed sertoliform/granulosa-like features) and should be distinguished from other entities such as neuroendocrine neoplasms, Wolffian/mesonephric-like tumors, and *STK11* adnexal tumors. Eosinophilic changes with nuclear pleomorphism can also be observed. The typical immunohistochemical pattern of SCLEC includes positivity for CK7, EMA, and hormone receptors; most cases in our series also showed nuclear accumulation of β-catenin, CDX2 positivity, together with a CD10 pattern that might mimic Wolffian/mesonephric-like tumors. Sex cord markers, neuroendocrine markers, and mesonephric markers GATA3 and TTF1 were negative instead. Although most SCLECs in our study were NSMP and appeared similar to low-grade ECs, four cases showed high-grade nuclei and one showed p53-abnormal pattern and BRCA mutation, suggesting that SCLEC is a heterogeneous entity. Given the rarity of SCLEC and the relatively small sample size of the published studies, further research appears necessary in this field.

## Data Availability

Additional data are available from the corresponding author upon reasonable request.

## References

[1] Malpica A (2016) How to approach the many faces of endometrioid carcinoma. Mod Pathol 29(1):S29-44 26715172 10.1038/modpathol.2015.142

[2] Young RH, Prat J (1982) Scully RE (1982) Ovarian endometrioid carcinomas resembling sex cord-stromal tumors. A clinicopathological analysis of 13 cases. Am J Surg Pathol. 6:513–22 6890771 10.1097/00000478-198209000-00003

[3] Roth LM, Liban E, Czernobilsky B (1982) Ovarian endometrioid tumors mimicking Sertoli and Sertoli-Leydig cell tumors: sertoliform variant of endometrioid carcinoma. Cancer 50(7):1322–1331 7104975 10.1002/1097-0142(19821001)50:7<1322::aid-cncr2820500718>3.0.co;2-c

[4] Eichhorn JH, Young RH, Clement PB (1996) Sertoliform endometrial adenocarcinoma: a study of four cases. Int J Gynecol Pathol 15(2):119–126 8786200 10.1097/00004347-199604000-00005

[5] Fujibayashi M, Aiba M, Iizuka E, Igarashi A, Muraoka M, Takagi K (2005) Granulosa cell tumor-like variant of endometrioid carcinoma of the ovary exhibiting nuclear clearing with biotin activity: a subtype showing close macroscopic, cytologic, and histologic similarity to adult granulosa cell tumor. Arch Pathol Lab Med 129(10):1288–1294 16196518 10.5858/2005-129-1288-GCTVOE

[6] Guerrieri C, Frånlund B, Malmström H, Boeryd B (1998) Ovarian endometrioid carcinomas simulating sex cord-stromal tumors: a study using inhibin and cytokeratin 7. Int J Gynecol Pathol 17(3):266–271 9656124 10.1097/00004347-199807000-00012

[7] Zhao C, Bratthauer GL, Barner R, Vang R (2007) Comparative analysis of alternative and traditional immunohistochemical markers for the distinction of ovarian sertoli cell tumor from endometrioid tumors and carcinoid tumor: a study of 160 cases. Am J Surg Pathol 31(2):255–266 17255771 10.1097/01.pas.0000213355.72638.f4

[8] Bennett JA, Oliva E (2022) The complex and often confusing history, histology and histogenesis of mesonephric, STK11 adnexal tumour and mesonephric-like neoplasms of the upper female genital tract (including broad ligament). Histopathology 81(3):280–296 35395118 10.1111/his.14662

[9] D’Alessandris N, Travaglino A, Santoro A et al (2021) TCGA molecular subgroups of endometrial carcinoma in ovarian endometrioid carcinoma: a quantitative systematic review. Gynecol Oncol 163(2):427–432 34446267 10.1016/j.ygyno.2021.08.011

[10] Ciucci A, De Stefano I, Vellone VG et al (2013) Expression of the glioma-associated oncogene homolog 1 (gli1) in advanced serous ovarian cancer is associated with unfavorable overall survival. PLoS ONE 8(3):e60145 23555905 10.1371/journal.pone.0060145 PMC3610749

[11] Travaglino A, Raffone A, Arciuolo D et al (2022) Diagnostic accuracy of HNF1β, napsin A and P504S/alpha-methylacyl-CoA racemase (AMACR) as markers of endometrial clear cell carcinoma. Pathol Res Pract 237:154019 35907281 10.1016/j.prp.2022.154019

[12] Travaglino A, Arciuolo D, Santoro A et al (2023) Corded and hyalinized endometrioid endometrial carcinoma with high-grade features: a clinicopathological and TCGA-based molecular analysis. Virchows Arch 482(4):671–678 36550216 10.1007/s00428-022-03472-8

[13] Nofech-Mozes S, Ghorab Z, Ismiil N et al (2008) Endometrial endometrioid adenocarcinoma: a pathologic analysis of 827 consecutive cases. Am J Clin Pathol 129(1):110–114 18089496 10.1309/UDYANQ6XTK6UUTXQ

[14] Dridi M, Chraiet N, Batti R et al (2018) Granulosa cell tumor of the ovary: a retrospective study of 31 cases and a review of the literature. Int J Surg Oncol 29(2018):4547892 10.1155/2018/4547892 PMC5896205 29796312

[15] Young RH, Scully RE (1985) Ovarian Sertoli-Leydig cell tumors A clinicopathological analysis of 207 cases. Am J Surg Pathol 9(8):543–69 3911780 10.1097/00000478-198508000-00001

[16] Ordi J, Schammel DP, Rasekh L, Tavassoli FA (1999) Sertoliform endometrioid carcinomas of the ovary: a clinicopathologic and immunohistochemical study of 13 cases. Mod Pathol 12(10):933–940 10530556

[17] World Health Organization (2020) Classification of tumours editorial board. Female genital tumours. WHO classification of tumours series, 5th edn, vol 4. International Agency for Research on Cancer, Lyon

[18] Rindi G, Mete O, Uccella S et al (2022) Overview of the 2022 WHO classification of neuroendocrine neoplasms. Endocr Pathol 33:115–154 35294740 10.1007/s12022-022-09708-2

[19] Bennett JA, Ritterhouse LL, Furtado LV et al (2020) Female adnexal tumors of probable Wolffian origin: morphological, immunohistochemical, and molecular analysis of 15 cases. Mod Pathol 33(4):734–747 31591497 10.1038/s41379-019-0375-9

[20] Pors J, Cheng A, Leo JM, Kinloch MA, Gilks B, Hoang L (2018) A Comparison of GATA3, TTF1, CD10, and calretinin in identifying mesonephric and mesonephric-like carcinomas of the gynecologic tract. Am J Surg Pathol 42(12):1596–1606 30148742 10.1097/PAS.0000000000001142

[21] Bennett JA, Young RH, Howitt BE et al (2021) A Distinctive adnexal (usually paratubal) neoplasm often associated with Peutz-Jeghers syndrome and characterized by STK11 alterations (STK11 adnexal tumor): a report of 22 cases. Am J Surg Pathol 45(8):1061–1074 33534223 10.1097/PAS.0000000000001677 PMC8277663

[22] Travaglino A, Raffone A, Russo D et al (2021) Does endometrial morular metaplasia represent odontogenic differentiation? Virchows Arch 479(3):607–616 33666744 10.1007/s00428-021-03060-2 PMC8448715

[23] Travaglino A, Arciuolo D, Santoro A et al (2023) Corded and hyalinized endometrioid carcinoma: summary of clinical, histological, immunohistochemical and molecular data. Pathol Res Pract 247:154515 37209572 10.1016/j.prp.2023.154515

[24] Xu J, Park KJ, Weisman PS (2023) An expanded series of Pilomatrix-like high-grade endometrioid carcinoma (PiMHEC), including both MMR deficient and MMR proficient cases. Int J Gynecol Pathol 43(1):67–69. 10.1097/PGP.0000000000000955 10.1097/PGP.0000000000000955 PMC10625643 37192144

[25] Santoro A, Travaglino A, Valente M et al (2022) Pilomatrix-like high-grade endometrioid carcinoma of the ovary: case report, literature review, and differential diagnosis. Diagnostics (Basel) 12(12):3146 36553153 10.3390/diagnostics12123146 PMC9777718

[26] Wani Y, Notohara K, Nakatani Y, Matsuzaki A (2009) Aberrant nuclear Cdx2 expression in morule-forming tumours in different organs, accompanied by cytoplasmic reactivity. Histopathology 55(4):465–468 19817898 10.1111/j.1365-2559.2009.03382.x

[27] Malmros K, Lindholm A, Vidarsdottir H et al (2023) Diagnostic gastrointestinal markers in primary lung cancer and pulmonary metastases. Virchows Arch. 10.1007/s00428-023-03583-w 10.1007/s00428-023-03583-w PMC11329406 37349623

[28] Suh E, Traber PG (1996) An intestine-specific homeobox gene regulates proliferation and differentiation. Mol Cell Biol 16(2):619–625. 10.1128/MCB.16.2.619 8552090 10.1128/mcb.16.2.619 PMC231041

[29] Werling RW, Yaziji H, Bacchi CE, Gown AM (2003) CDX2, a highly sensitive and specific marker of adenocarcinomas of intestinal origin: an immunohistochemical survey of 476 primary and metastatic carcinomas. Am J Surg Pathol 27(3):303–310. 10.1097/00000478-200303000-00003 12604886 10.1097/00000478-200303000-00003

[30] Karnezis AN, Aysal A, Zaloudek CJ, Rabban JT (2013) Transitional cell-like morphology in ovarian endometrioid carcinoma: morphologic, immunohistochemical, and behavioral features distinguishing it from high-grade serous carcinoma. Am J Surg Pathol 37(1):24–37 23108017 10.1097/PAS.0b013e31826a5399

[31] Madore J, Ren F, Filali-Mouhim A et al (2010) Characterization of the molecular differences between ovarian endometrioid carcinoma and ovarian serous carcinoma. J Pathol 220(3):392–400 19967725 10.1002/path.2659

[32] Travaglino A, Santoro A, Arciuolo D et al (2023) High-grade serous ovarian carcinoma with a sertoliform pattern associated with BRCA mutation: a clinicopathological and molecular analysis. Virchows Arch 483(6):879–883. 10.1007/s00428-023-03556-z 10.1007/s00428-023-03556-z 37166561

